# Hospital and Institutional News

**Published:** 1916-04-08

**Authors:** 


### HOSPITAL AND INSTITUTIONAL NEWS.

### THE HOSPITAL SHIP "PORTUGAL."

An outrage upon a hospital ship by the Huns was, of course, bound to come in time, but one certainly expected that when it did happen it would be contrived with more regard for appearances and more opportunity for subterfuges than in the case of the unfortunate *Portugal*. This ship, properly notified to and recognised by the enemy months ago as a hospital ship, properly painted and distinguished as required by the Geneva Convention, flying the proper Red Cross flag, was torpedoed in broad daylight whilst at anchor, or just about to anchor, off the Anatolian coast. One account from Petrograd states that the submarine circled all round the ship and then discharged two torpedoes at 60 yards' range. The ship sank in less than a minute, and only the presence of a good many boats close to the scene of the outrage was the means of saving 158 out of the 273 souls on board. In endeavouring to probe the motives of this abominable crime one is confronted with the usual difficulty of plumbing the deeps of German insanity. Can it be that the name of the ship offended German eyes on account of the accession of Portugal to the Allied cause?

### LONDON COUNTY COUNCIL WISDOM.

The decision of the London County Council to undertake the inspection of lying-in homes throughout the county of London, and not to delegate the duty to the Metropolitan Borough Councils, is momentous for good. The fact that the amendment to delegate the duty to Borough Councils should have been moved by a Mr. Norman, whom the reporter describes as "the leader of the Municipal Reform party," is eloquent in its testimony to the soundness of an ever-extending public opinion, to the effect that it will be well for London when Borough Councils are abolished, with all the ancient abuses, inefficiencies, and evils which they have inherited from vestry and Metropolitan Board days. The effort of the Municipal Reform party's policy in this instance was shown in the course of the discussion to mean that inspection by a Borough Council meant (1) an inspector of nuisances, (2) that the worst class of lying-in home was evading inspection by moving into the next borough, and (3) that the Borough Councils, if this inspection was given to them, did not anticipate any extra expense or addition to their staff. In other words the Municipal Reformer's ideal is to rely upon ignorance and to block progress. Party names are usually meaningless as indicators of principles, and the metropolitan ratepayers will do well to remember the illuminating light the discussion in the London County Council on the 4th instant on maternity homes has thrown upon the dangers attending the admission of members of the Municipal Reform party to seats on the County Council, or, indeed, on any body which has to do with the life, the hygienic surroundings, the protection and happiness of poorer Londoners. Is it too much to hope that members of Parliament will during the present war—a period of luxurious leisure to the majority of M.P.s—spend a little time in studying the underlying dangers of longer permitting Borough Councils to exist within the metropolitan area. Such a task should prove illuminating, assuming that Mr. Norman is in fact the leader of "the Municipal Reform party," and that his action at the London County Council meeting on Tuesday accurately represents its policy.

### THE CITY CORONER'S REPORT.

The annual return which the Coroner for the City and for Southwark renders to the Corporation of the City of London deals this year with many topics which affect institutions; such as fatalities under anæsthesia; casualties due to darkened streets, the strength of laudanum, and so forth. As regards street accidents, he makes some pointed remarks, with which experienced drivers of horse or motor vehicles will fully agree, on the "careless disregard of pedestrians for their own personal safety." Several elementary rules of prudent conduct when crossing a road are mentioned as being violated with exceptional frequency, and several recommendations for the numerical reduction of traffic accidents are put forward. Another interesting paragraph is that which deals with a find of Tudor ornaments and jewellery alleged to have been made in the City. According to statute, such finds become the property of the Corporation after inquest by the Coroner to determine whether or no they are "treasure trove." In this case the hoard is stated to have been secretly removed from the City and to have been "given" to the London Museum and to the British Museum by the Right Hon. Lewis Harcourt. The Coroner also extols the value of the Herscher formalin apparatus for preserving unidentified bodies: his experience with it is so satisfactory that he would like to see it more generally installed in Coroners' mortuaries.

### A COAL SEAM UNDER A HOSPITAL.

The Lord Mayor of Leeds (Mr. Charles Lupton) has been for so long a pillar of hospital organisation in that city that his address at the annual meeting of the Leeds Infirmary was naturally more than usually interesting and informative. The work on the King Edward Memorial Extension Buildings is proceeding satisfactorily; the nurses' home extension is already occupied, and the ward block, the operating theatres, and the out-patient block are well advanced. In connection with the new boiler-house difficulty was encountered owing to the presence of a seam of coal, which had to be removed before the work could proceed. Mr. Lupton did not refer to this seam as a source of profit, so probably it is not workable; but if it were, its presence under the boiler-house would be most convenient! The financial situation is by no means satisfactory; this Mr. Lupton attributes to the fact that "the city and county do not realise the magnitude of the work done at the infirmary and their responsibilities towards it." There is an adverse balance for the year of nearly £7,000, in spite of the fact that legacies yielded £6,000 more than in 1914. This is a state of things which surely cannot be satisfactory to a city like Leeds. Mr. Lupton has indicated the remedies, and it is for the citizens and workers of Leeds to pay heed to his admonitions.

### HOSPITALS AND THE BAN ON GLYCERINE.

The policy of the Ministry of Munitions in requiring manufacturers of glycerine to withhold supplies from chemists unless they give a written undertaking to sell glycerine only when it is ordered by a medical practitioner and not to use it as an ingredient of toilet preparations is not likely to affect hospitals to any considerable extent. It will be the duty of prescribers, however, to refrain from ordering glycerine except in cases where it is quite necessary, and hospital dispensers should at once take steps to inform the medical staff of suitable substitutes for glycerine in preparations both for internal and external use. Under no circumstances should glycerine be used merely as a sweetening agent. Whether or not hospital authorities receive an official notice requiring them to use glycerine as economically as possible, they should, without loss of time, issue instructions that this should be done. In the aggregate the quantity of glycerine used medicinally is very large, and it is essential that it should be reduced, since the requirements of this article for the purpose of manufacturing explosives are steadily growing.

### THE WOMAN'S IMPERIAL HEALTH ASSOCIATION

The sixth annual report of this Association deals with the busiest year yet experienced, notwithstanding the curtailment by the war of some of the propaganda—*e.g.* the caravan tours. More than nine-tenths of the income of the Association is derived from cinematograph theatres by permission of the London County Council (presumably from Sunday performances), and about 2 per cent. of it is obtained from subscriptions. It is clear that the income is being wisely and well expended; and, inasmuch as there was a deficit for the year of £130, it would seem on the face of matters that a vigorous effort to lengthen the subscription-list is one of the needs of the moment. Mothercraft exhibitions and conferences, juvenile health crusades, leaflets, lectures, infant weighings, almoner's work at lying-in hospitals, and many other beneficent undertakings are included among the activities of the Association.

### "PTOMAINE" POISONING.

In the *Western Morning News* of March 28 is the report of inquests on the deaths of two boys (brothers), who it was alleged died of ptomaine poisoning from consuming "tinned" milk. The medical evidence was that one child, aged four years, was dead when the doctor arrived, and the other, aged seven years, was found to be dangerously ill and died next day. It was also stated that the mother was lying dangerously ill suffering from gastro-enteritis. The father is a sergeant with his regiment in Egypt. That the unfortunate patients suffered from irritant poisoning is fairly evident from the short report, but that the symptoms were caused by a ptomaine is not at all clear. Ptomaines are not often found in sweetened condensed milk. There was also a suggestion that the symptoms were due to bacterial infection, but at this time of the year, when no flies are about, microbic contamination of food is at its minimum. Gaertner's bacilli and *Bacillus botulinus* infections do not as a rule result from condensed milk. No analysis nor bacteriological examinations of the contents of the stomachs and intestines nor the milk were made. The result of the inquiries, even in war-time, must be considered unsatisfactory. In the same paper of March 29 is the report of an inquest on a little girl aged five years, who is also alleged to have died of ptomaine poisoning. The evidence was that she complained of pain in the neck. The report does not say if she partook of any food, nor what there was for dinner; but it was suspected that she took something from the dustbin in the yard. She vomited several times, and her temperature rose; she died the next day. It was stated that her sister, aged three years and eleven months, died on March 3 after twenty hours' illness. A *post-mortem* examination was made, "but as no food was found in the stomach no analysis could be made." Either fulminating scarlet fever *sine eruptione* or cerebrospinal fever would account for these cases much more probably than ptomaine poisoning.

### THE OXFORDSHIRE TUBERCULOSIS SCHEME HELD UP.

An unfortunate hitch has arisen in the negotiations which are proceeding between the Oxfordshire Tuberculosis Association and the County Council in connection with the inauguration of a complete tuberculosis scheme for the whole county. The Tuberculosis Association, of which Sir William Osler is the president, offered to transfer its staff, dispensaries, shelters, and other equipment to the County Council, and the local Insurance Committee was also preparing to enter into a monetary agreement with the Council for the institutional treatment of insured persons. The transference of the assets of the voluntary Association, with its medical and nursing staffs, was apparently about to be accepted by the County Council at a recent meeting, especially as the Association was willing to continue that part of its work which dealt with after-care. The present officer of the Association, Dr. Stobie, was to be the clinical tuberculosis officer at a salary of £500, and Dr. Charles Coles, the county medical officer of health, was to be appointed chief tuberculosis officer at a salary of £150. At the above-mentioned meeting a letter from Sir William Osler was read, in which he asked, on behalf of the Association, for the appointment of a sub-committee to supervise the relations between the whole-time tuberculosis officer and his chief, the county medical officer. It is understood that the Association has grounds for believing Dr.

Coles to be hostile to the dispensary system, and it does not view with favour the prospect of working with him as chief tuberculosis officer, though it has every confidence in him as an administrator. The suggestion for the sub-committee is in the nature of a safeguard for the Association. After the reading of this letter, however, the Council decided that the scheme should be referred back for three months. It is expected that the County Council will submit a scheme to the Tuberculosis Association, and that presently the whole matter will be satisfactorily arranged.

### THE GREAT NORTHERN CENTRAL HOSPITAL.

This year the Great Northern Central Hospital attains its diamond jubilee, an event which would in normal times evoke some special celebration. The unpropitious times, the Chairman (Mr. E. Smithett) announced at the annual council of governors lately held, render it difficult to celebrate the occasion; but every effort will be exerted, nevertheless, to place the institution on a firm financial basis. In spite of the serious rises in the price of provisions, drugs, and other necessaries, the cost per occupied bed has actually been reduced by nearly £3 a year during the last twelvemonth. An exceptionally good year in the way of legacies and other windfalls enabled ends to be met; but it is evident that, with an annual expenditure now amounting to over £24,000, there is a very heavy task before the management each year in respect of the raising of funds. Evidently, from the Chairman's speech, the responsibility is fully realised and is being faced with courage and cheerfulness. The right note has been struck; given energetic friends, the Great Northern should be able to meet the future with confidence and equanimity.

### LANCASTER INFIRMARY'S SUCCESS IN LITIGATION.

A disputed point in connection with certain bequests to the Lancaster Infirmary made by the late Mr. William Richmond, of Lancaster, has been decided in favour of the institution. The trustees of the will, uncertain of their exact responsibilities, applied to the Manchester Chancery Court for an interpretation of the will, in which the testator left his residence, "Slynesdale," for the establishment of a convalescent home, to be called the William Richmond Convalescent Home, in connection with the Lancaster Infirmary. The infirmary authorities did not take out a licence because they thought the house unsuitable for the purpose; and the contention of the other side was that this failure to accept the house disentitled them to other parts of the gifts under the will. The Vice-Chancellor held that the gifts included everything in the will, including the residuary personal estate; and he authorised the trustees to pay the proceeds of the sale of "Slynesdale," if sold, to the treasurer of the institution.

### A STATUE TO NURSE CAVELL.

It is announced in the *Daily Telegraph*, which is raising a fund for the purpose, that the memorial statue to Miss Cavell is arranged for, though it will be two years before it is completed. Sir George Frampton is devoting his whole time to the memorial, to which his work is his personal contribution; and the bust which he has made will be exhibited at this year's Academy. A site for the memorial has been given by the Westminster City Council and approved by the Office of Works; it is the island in the road opposite the National Portrait Gallery. The memorial will face south, and will be 33 feet in height and of obelisk shape. The figure of Edith Cavell, in nurse's uniform, will be of white marble, outlined against a silvery-grey granite column. The monument will be crowned by a seated figure of Justice and Humanity, with a sword across her knees, guarding an infant. At the back a lion, in white marble, stamps upon a serpent. The sculptor's sketch model of the monument is not being sent to the Academy.

### £3,000 IN TEN MINUTES.

They are great "hustlers" in Yorkshire, and have a reputation to live up to. The magnates of Keighley are plainly alive to both these facts, for at a recent meeting of about a score of gentlemen in the Mayor's Parlour—and the meeting was called for the election of a committee rather than for the raising of money—there was actually £3,000 put up in ten minutes. The scheme which elicited this display of generosity was one for adapting and extending the Morton Banks Hospital for the reception of 400 wounded soldiers. The Mayor (Mr. W. A. Brigg) explained the details of the scheme to the meeting, and mentioned that £7,000 would be needed to carry it through. Sir Berkeley Moynihan also addressed the meeting; and as he is perhaps the most finished and eloquent speaker in the medical profession at the present time, the instantaneous response may be placed largely to his credit without serious risk of error. By the time the meeting closed £3,330 had been promised, and a pledge of a further sum of between £600 and £1,000 was made on behalf of the Bingley district.

### URGENT REQUIREMENTS AT THE ESSEX COUNTY HOSPITAL, COLCHESTER.

The most important parts of the annual report of this institution and of the discussion at the annual meeting referred to the urgent needs of two departments—the radiographic and the ophthalmic. It appears that the work of the former has been carried out in much too small a room, with very inadequate equipment; and it is felt that the conditions constitute a distinct danger to the health of the medical officer who manages the department. The sum of £500 is required to provide an *x*-ray equipment adequate to the requirements of the hospital and to the needs of the soldier patients, and a similar sum is needed to provide and equip an ophthalmic department. At the moment, it seems, eye cases requiring specialist attention have to be sent away to Ipswich or to London, owing to the resignation of the medical officer who has hitherto undertaken this work. However, an ophthalmic specialist is stated to have just settled in Colchester who is willing to undertake the work if a properly equipped department is provided. There are minor requirements mentioned in the report, but these two departments appear to be the really pressing needs of the moment; and it is to be hoped some generous friends of the institution may be found who will render possible their satisfaction.

### SURGICAL OPERATIONS ON SCHOOL CHILDREN.

The Liverpool Education Committee at their last meeting discussed the advisability of establishing a clinic for the treatment of adenoids and enlarged tonsils at a cost of £995. In spite of an able speech by Dr. Moyles (chairman of the Medical Inspection of School Children Sub-Committee) in support of the recommendation, the members rejected the proposal by eighteen votes to eleven. This decision was arrived at on the grounds of the necessity for economy at the present time. It is to be regretted that the Education Committee of a large city should fail so completely to realise the importance of the health of the children. It is a trite saying, but one apparently requiring constant repetition, that the greatest asset of a nation is health. Money saved by the neglect of measures for improving the standard of well-being amongst the children will prove an expensive form of economy.

### THE AMBULANCE OF THE MAGHERAFELT GUARDIANS.

At the last meeting of the Magherafelt Guardians (Ireland) a letter was read from one of the members who stated that he was present when the ambulance was sent to fetch in a patient. On examining it he was astonished to find there was neither sheet nor pillow, only a dirty mattress and a still dirtier blanket. The relieving officer, when questioned, admitted the truth of these statements. The most amazing part of the matter was the condonation by the Chairman of this state of affairs. He is reported to have said that the vast majority of the people admitted were in a state of filth and dirt, and the Board need not be particular in sending out scrupulously clean clothes. After this one is not surprised to find the master of the workhouse offering as a refutation of the charges the statement that the clothing had been renewed THREE DAYS before the occurrence of which complaint was made. Most Englishmen would consider that the dirty condition of patients brought to the infirmary would be a strong argument in favour of a supply of fresh linen and blankets for every case, but perhaps the Magherafelt Guardians would regard this as merely Saxon stupidity.

### THE WOMAN'S REAL WORLD.

A glance at the doings of cottage hospitals tempts one to wonder whether they do not rival the larger institutions in the record of length of service on the part of their staffs. It is among them that women are frequently found, and every hospital manager knows that there is no more loyal and unflagging voluntary worker than a woman when, as frequently happens, she is one of the right sort. Cottage hospital presidents are usually working presidents, and women not infrequently fill these posts to the great advantage of the institutions. Again, as honorary secretaries of cottage hospitals and nursing associations their services are as remarkable as they are unobtrusive. But length of service is no monopoly of either sex. If women's sphere be really the home, it should be spelt with a capital "H," to signify an institution. For instance, the Market Harborough Cottage Hospital and Nursing Association has lately lost the services of Mrs. Jeffries, who has been for twenty-nine years honorary secretary and treasurer. The extent of her duties can be guessed from the fact that two people now divide the joint office between them. For the past two years the president of the hospital has been Mrs. Mills. Again, the Harrow Cottage Hospital has lately accepted the resignation of Mr. J. N. Stuart after seventeen years' service as honorary secretary, during part of which Mrs. Stuart has acted as superintendent of the lady collectors. The election of Mr. J. N. Stuart to the position of vice-president is a deserved tribute to his services.

### THIS WEEK'S DRUG MARKET.

Business has been rather quieter, but on the whole prices are well maintained. There are, however, a few exceptions, one of which is potassium bromide, makers' quotation for which has been very slightly reduced; the values of ammonium bromide and sodium bromide are unchanged. There is no substantial change in the position of cod-liver oil, and the extremely high prices continue to keep buyers off the market. Tartaric acid and citric acid are still very scarce and fetching very high prices. Formaldehyde is again dearer. Hexamine has an upward tendency in value. Sulphonal is rather lower. Balsam copaiba is slightly dearer. The advance in the price of potassium permanganate continues. Castor oil has a slightly lower tendency in price. Epsom salts may probably be dearer. The price of glycerine is unchanged, but it is very difficult to obtain supplies, buyers being required to give an undertaking to dealers that any glycerine supplied shall only be used for *bona-fide* medicinal purposes. The high value of cocaine is well maintained. A considerable advance in the prices of rectified spirit and methylated spirit has taken place, owing to the shortage of raw material and the increased demand for spirit for the manufacture of explosives; in consequence, the prices of tinctures and all spirituous preparations will be raised. The increase in the sugar-tax will only affect the cost of manufacture of medicinal syrups and other compounds containing sugar to a trifling extent.

### TO OUR READERS.

**Contributions are specially invited from any of our readers to these columns. They should deal with topical subjects and news. They must be authenticated for the information of the Editor only. The minimum payment if published is 5s. There is no hard-and-fast rule as to space, but notes of about twenty lines in length are preferred.**

